# Relationship Between Sarcopenia and Cardiovascular Diseases in the Elderly: An Overview

**DOI:** 10.3389/fcvm.2021.743710

**Published:** 2021-12-09

**Authors:** Nana He, Yuelin Zhang, Lu Zhang, Shun Zhang, Honghua Ye

**Affiliations:** ^1^Department of Cardiology, HwaMei Hospital (Previously Named Ningbo No. 2 Hospital), University of Chinese Academy of Sciences, Ningbo, China; ^2^Department of Experimental Medical Science, HwaMei Hospital, University of Chinese Academy of Sciences, Ningbo, China; ^3^Key Laboratory of Diagnosis and Treatment of Digestive System Tumors of Zhejiang Province, Ningbo, China; ^4^Department of Medicine, University of Ningbo, Ningbo, China

**Keywords:** sarcopenia, cardiovascular diseases, elderly people, comorbidity, aging

## Abstract

With the advent of population aging, aging-related diseases have become a challenge for governments worldwide. Sarcopenia has defined as a clinical syndrome associated with age-related loss such as skeletal muscle mass, strength, function, and physical performance. It is commonly seen in elderly patients with chronic diseases. Changes in lean mass are common critical determinants in the pathophysiology and progression of cardiovascular diseases (CVDs). Sarcopenia may be one of the most important causes of poor physical function and decreased cardiopulmonary function in elderly patients with CVDs. Sarcopenia may induce CVDs through common pathogenic pathways such as malnutrition, physical inactivity, insulin resistance, inflammation; these mechanisms interact. In this study, we aimed to investigate the relationship between sarcopenia and CVDs in the elderly. Further research is urgently needed to understand better the relationship, pathophysiology, clinical presentation, diagnostic criteria, and mechanisms of sarcopenia and CVDs, which may shed light on potential interventions to improve clinical outcomes and provide greater insight into the disorders above.

## Introduction

Sarcopenia is a progressive and widespread decline in skeletal muscle mass and function, leading to the loss of workforce and mobility in the elderly. The onset and progression of sarcopenia are closely associated with old age, skeletal muscle disuse, malnutrition, chronic systemic inflammation, and anabolic disorder. Sarcopenia puts a great deal of pressure on society by significantly increasing hospitalization and mortality rate in elderly patients (1, 2). Sarcopenia is a relatively common disease that causes impaired exercise capacity in the elderly, strongly associated with CVDs. Aging and dysfunction of the locomotor system limit exercise in the elderly, increasing the risk of CVDs. CVDs and sarcopenia can coexist, further reducing exercise tolerance and quality of life and increasing mortality. Sarcopenia and CVDs interact to accelerate the disease process (3, 4). As the global population ages, the number of patients with CVDs and sarcopenia increases, and the resulting health problems such as loss of work capacity and mobility in the elderly are of concern. Exploring the mechanism between sarcopenia and CVDs could provide a scientific basis for clinical diagnosis and treatment.

## Sarcopenia

### Definition

Derived from the Greek words sarx (muscle) and penia (deficiency), sarcopenia is a chronic degenerative senile syndrome similar to osteoporosis. It has a significant impact on the quality of life of the elderly. Sarcopenia was first introduced by Rosenberg in 1989 and refers to age-related muscle loss and muscle strength decline (5). Initially, the understanding of “sarcopenia” was limited to reducing skeletal muscle mass with age in older people. Subsequently, it was discovered that the muscle mass of skeletal muscles is not linearly related to muscle strength and function and that a decline in pure muscle mass is not significantly associated with functional status in older people. For a long period of time, research on sarcopenia did not make much progress. It was not until 2010 (6) that the European Working Group on Sarcopenia in Older People (EWGSOP) first proposed a clinical definition of sarcopenia. It is recommended that both skeletal muscle mass and function (muscle strength or mobility) be reduced for a diagnosis of sarcopenia to be made. The definition of sarcopenia also changed from the initial decades of focusing only on the muscle mass of skeletal muscle to focus on both a decrease in muscle mass and muscle strength and more on changes in function. In 2011 (7), the International Working Group on Sarcopenia (IWGS) provided a similar definition of sarcopenia, emphasizing physical function evaluation, including the ability to sit up from a chair or a pace test. In 2014 (8), the Asia Working Group for Sarcopenia (AWGS) and the Foundation for the National Institutes of Health (FNIH) also launched their respective expert consensus on sarcopenia. In 2018 (9), the EWGSOP, based on the latest results of basic and clinical research on sarcopenia in the past 10 years, renewed its consensus. EWGSOP defines sarcopenia as a syndrome of progressive and generalized decline in skeletal muscle mass associated with low muscle strength or low physical performance. In addition, the EWGSOP newly identifies subcategories of sarcopenia as acute and chronic. In addition, sarcopenia lasting for <6 months is considered an acute disease, while those with an illness for more than six months are considered a chronic disease. In 2019 (10), the AWGS also updated its consensus on sarcopenia. Sarcopenia is age-related loss of muscle mass associated with low muscle strength and or low physical performance. The EWGSOP definition of sarcopenia is now widely used internationally. Sarcopenia is associated with various adverse outcomes, including falls, dysfunction, weakness, and death. Sarcopenia is now officially recognized as a muscle disorder with the diagnosis code ICD10-MC, suggesting that it will receive widespread attention from the medical community as a disorder with unique characteristics and a more accurate understanding of screening, diagnosis, intervention, and treatment of the condition (11).

### Diagnosis

Due to the lack of specific clinical manifestations of sarcopenia and the fact that human muscle mass is affected by various factors such as race, region, age, and gender, there is currently no unified standard for diagnosing sarcopenia at home and abroad. EWGSOP (9), AWGs (10), IWGS (7), FNIH (12) all state that the diagnosis of sarcopenia should take into account a combination of muscle mass and muscle function, with the leading indicators including muscle mass, muscle strength, and muscle function. Specific updated diagnostic criteria for sarcopenia are shown in Table 1. The prevalence of sarcopenia varies with different diagnostic criteria and different measures of muscle mass. The prevalence of sarcopenia in the elderly in the community ranges from 8.7 to 28.5% under different diagnostic criteria (13).

**Table 1:** The specific latest diagnostic criteria for sarcopenia.

**Criterion**	**Muscle mass**	**Muscle strength**	**Muscle function**
EWGSOP2	ASM/height^2^ <7.0 kg/m^2^ in men ASM/height^2^ <6.0 kg/m^2^ in women	Handgrip<27 Kg in men Handgrip<16 Kg in women	Walking speed≤0.8 m/s
AWGS2	ASM/height^2^ <7.0 kg/m^2^ in men ASM/height^2^ <5.4 kg/m^2^ in women	Handgrip<28 Kg in men Handgrip<18 Kg in women	Walking speed<1.0 m/s
IWGS	ASM/height^2^ <7.23 kg/m^2^ in men ASM/height^2^ <5.67 kg/m^2^ in women	-	Walking speed<1.0 m/s
FNIH	ASM/BMI <0.789 in men ASM/BMI <0.512 in women	Handgrip<26 Kg in men Handgrip<16 Kg in women	Walking speed≤0.8 m/s

### Epidemiological Characteristics

Epidemiological studies have found that skeletal muscle begins to degenerate after the 40 and accelerates with age. The quantity and quality of skeletal muscle are declining at a rate of ~8% per year (14). Due to differences in study populations, research methods and diagnostic criteria, the prevalence of sarcopenia varies greatly between different studies. The prevalence of sarcopenia in people over 50 years of age ranges from 1–33% (11). In particular, the prevalence of sarcopenia ranges from 5 to 13% in people aged 60 to 70 years and up to 50% in people aged 80 years and older (15). There are also significant differences in the incidence of sarcopenia in different regions. The prevalence of sarcopenia is 38.9% in males and 17.8% in females in Canada (16). In Australia, the prevalence of sarcopenia is <20% in the population aged 70 years or older (17). In the UK, the prevalence of sarcopenia in men and women is 4.6 and 7.9%, respectively (18). Research on sarcopenia in the Asian population is still in its infancy. In Asia, the incidence of sarcopenia in Thailand was 35.33% in men and 34.74% in women, respectively; in Japan, it was 6.7~11.3% in men and 6.3~11.7% in women; in South Korea, it was 6.3~21.8% in men and 4.1~22.1% in women; in China, it was 12.3% in men and 7.6% in women (8). The study included 200 elderly inpatients with an average age of 74.49 ± 6.32 years. Sarcopenia was detected in 28 (14%) of the patients. Among them, the in-hospital mortality rate of patients with sarcopenia was 28.6%, and that of patients without sarcopenia was 11.0%. There is an increased length of stay and mortality in older inpatients with sarcopenia (1). Differences in the prevalence of sarcopenia may be related to ethnicity, lifestyle, exercise habits, and the use of quantitative diagnostic criteria adopted by different research institutes. It is estimated that by 2050, there will be more than 200 million elderly patients with sarcopenia in the world (6). Sarcopenia can increase the risk of weakness, falls and fractures, decrease the quality of life, even the ability to live independently, and increase the infection rate, and mortality rate in patients. Sarcopenia is associated with CVDs, diabetes, renal insufficiency, cancer, cognitive impairment, and even with the prognosis of some diseases (19–22).

## Sarcopenia and CVDs

The main feature of sarcopenia is skeletal muscle disorders such as loss of muscle mass, quality, strength, and physical performance. It is commonly seen in elderly patients with chronic diseases. Sarcopenia may be considered one of the most important causes of poor physical performance and reduced cardiorespiratory fitness in older patients with CVDs. CVDs may induce sarcopenia through common pathogenetic pathways such as hormonal changes, malnutrition, and physical inactivity, mechanisms that influence each other. Sarcopenia is also an age-related disease closely related to CVDs, and there are similarities between the two in terms of risk factors and pathogenesis.

### Sarcopenia and Heart Failure (HF)

The prevalence of sarcopenia is high in older HF patients, with sarcopenia also predictor of HF prognosis. Patients with HF are often associated with decreased muscle and strength. The ubiquitin protease system, myogenic protein signaling pathways, apoptosis, malnutrition due to gastrointestinal edema, and inflammatory factors may all contribute to sarcopenia (23, 24). The impaired exercise tolerance of HF patients is related to the changes of failed cardiomyocytes and skeletal muscle cells. The onset, development, and progression of sarcopenia follow the same clinical course as HF, with the two interacting. One SICA-HF study covered 200 HF patients, with the average age of the patients was (70.8 ± 8.3) years. It was showed a prevalence of sarcopenia of 19.5% in patients with HF (25). Another study reported that 19.7% of HF patients with preserved ejection fraction have sarcopenia (26).

The coexistence of sarcopenia and HF may be the result of their common pathophysiological pathways. Skeletal muscle in patients with HF has multiple histological abnormalities, and 2/3 of patients with chronic heart failure (CHF) have myofibrillar atrophy and decreased muscle capillary density (27). Moreover, few studies have shown that the observable skeletal muscle fiber atrophy rate observable in patients with CHF is ~68% (28). The imbalance of muscle protein synthesis and decomposition is a major factor in the development of sarcopenia (29). Oxidative stress can accelerate skeletal muscle degeneration and increase muscle protein decomposition. Levels of inflammatory markers tend to be elevated in patients with HF. Studies have shown that high levels of inflammatory cytokines are negatively associated with muscle strength and mass (30). In patients with heart failure and sarcopenia, the level of growth hormone (GH) is increased, while the level of insulin-like growth factor-1(IGF-1) is significantly reduced, suggesting that there may be GH resistance, leading to an inhibition of skeletal muscle formation. In patients with HF, the PI3K/Akt/mTOR signaling pathway involved in regulating protein synthesis is inhibited, while the ubiquitin-protease system that promotes protein breakdown, autophagy, and apoptosis are overactivated, and the dynamic balance between skeletal muscle production and destruction is broken, and then sarcopenia (31). In addition, patients with HF may suffer from poor appetite and malabsorption due to urinary difficulties, nausea, adverse drug reactions, which leads to inadequate or excessive nutrient loss and gastrointestinal symptoms and is associated with the pathogenesis of sarcopenia. Reduced peripheral perfusion due to left ventricular insufficiency from HF and reduced physical activity, which limits daily activities, can also cause a reduction in skeletal muscle, leading to the development of sarcopenia (30). Paradoxically, sarcopenia is not associated with a sarcopenia cardiac muscle, but the cardiac muscle shows hypertrophy which seems to be “not-functional.” Physiological cardiac hypertrophy usually occurs during pregnancy and in athletes, while pathological hypertrophy induces by factors such as prolonged and abnormal hemodynamic stress (i.e., hypertensive state), which can lead to cardiac dysfunction. The cardiac mass modification and dysfunction process, called “cardiac sarcopenia,” is similar to what happens in skeletal muscle, but few current studies exist. In a FLEAR study of elderly hospitalized patients, it was found that 19.4% of patients had sarcopenia and HF; in the absence of sarcopenia, the prevalence of HF was 16.3%. Through an echocardiographic study, it was discovered a correlation between sarcopenia and cardiac hypertrophy (32). Previous studies have shown a negative correlation between grip strength and heart mass in patients at risk of sarcopenia. More importantly, the decrease of muscle strength is associated with the increase of ventricular mass and the reduction of ejection fraction, resulting in “not-functional cardiac hypertrophy” (33–35). Heart failure with preserved ejection fraction (HFpEF) represents an important cardiovascular entity with increasing prevalence and relatively high mortality. Therefore, the earliest description of HFpEF is mainly conceptualized as a diastolic filling disorder. Only later inflammation and multimorbidity, which play a key role in the development of sarcopenia, are considered the main factors in developing HFpEF (36, 37). In this case, a sarcopenia heart characterized by a “not-functional hypertrophy” may be considered as an intriguing hypothesis. Thus it can be seen that the interaction and mechanism between sarcopenia and HF are very complicated.

### Sarcopenia and Hypertension

At present, there are few studies on the relationship between sarcopenia and hypertension. A total of 1,611 Chinese elderly people aged ≥60 years, who were diagnosed and assessed according to the AWGS recommended algorithm, had been included in a study on the relationship between sarcopenia and cardiovascular risk factors (CVRF), including diabetes, hypertension, and dyslipidemia, was analyzed. The results showed that the high prevalence of sarcopenia in the Chinese elderly population is related to CVRF. In addition, diabetes and hypertension, rather than dyslipidemia, were significantly associated with sarcopenia. It indicated that CVRF, especially diabetes and hypertension, may help predict the risk of sarcopenia in the elderly (4). Some studies put forward the concept of “sarcopenia obesity,” specifically referring to the coexistence of sarcopenia and fat accumulation. A follow-up study of 3,320 people in Korea found that the 10-year risk of CVDs in obese patients with sarcopenia was higher than those with non-obesity and non-sarcopenia. In contrast, the 10-year risk of CVDs in patients with simple obesity or sarcopenia was not significantly increased. A British study followed up 4,252 older men and found that patients with sarcopenia obesity highly correlated with CVD mortality. Patients with sarcopenia obesity have a higher mortality rate, but the study did not observe an increase in CVD incidence. Studies have also shown that patients with sarcopenia obesity are at high risk of developing type 2 diabetes mellitus, hypertension, and hyperlipidemia (38–40). In the early stage, it was considered that sarcopenia caused by aging is related to an increased incidence of hypertensive retinopathy and hypertensive kidney damage. More recently, it has been found that hypertension is related to the decrease in the number of capillaries around muscle cells (41). Therefore, it has been hypothesized that blood pressure-induced changes in the capillary network of muscle tissue are one of the risk factors for the occurrence of sarcopenia in elderly patients.

### Sarcopenia and Atherosclerosis

Atherosclerotic cardiovascular diseases (ACVDs), such as coronary atherosclerotic heart disease, atherogenic stroke or transient ischemic attack, transient ischemic attack, and peripheral artery disease, are acute diseases that affect the health of older people. They are also a direct cause of death, disability, and high medical costs. Exploring the related comorbidities and risk factors, looking for reliable prognostic markers has become a current research hotspot. A study of 335 Japanese subjects (mean age 64.9 years) found that risk factors for atherosclerosis (blood pressure, cholesterol) were significantly higher in the sarcopenia group than in the control group (*P* < *0.05*), after controlling for age, gender, and body mass index (BMI) (42). A large-scale cohort study showed that early atherosclerosis index carotid-femoral pulse wave velocity (PWV) independently and negatively correlated with skeletal muscle mass in Americans aged 70 to 79 (43). The decline in muscle mass and muscle strength was associated with endothelial dysfunction in another study of 208 Brazilian people over 80 years of age, with a 3.6-fold increased risk of atherosclerosis due to a decrease in muscle mass, suggesting that sarcopenia is strongly associated with atherosclerosis. This correlation was also found in middle-aged people, and the results were more precise in middle-aged men (44). A cross-sectional study of 31,108 middle-aged Koreans found that relative limb skeletal muscle index (RSMI) linearly correlated with the prevalence of coronary heart disease (CHD) and coronary artery calcification (CAC) score. After excluding insulin resistance or lack of physical activity, low muscle mass remained an independent risk factor for CHD (45). In addition, another study used ultrasound to measure the intima-media thickness (IMT) in 321 patients with ischemic heart disease to determine arteriosclerosis and used an index of muscle function as a criterion to determine sarcopenia. The carotid artery thickness is divided into two groups (IMT ≤ 2.6 mm) and low (MT > 2.6 mm). The results show a significant correlation between the lower limb muscle function index (step speed) and the isometric strength of the quadriceps in patients with ischemic heart disease and arteriosclerosis. In contrast, the grip strength does not correlate with it (46). Therefore, it can be concluded that reducing skeletal muscle is an independent risk factor for arteriosclerosis vascular disease and is closely related to other risk factors of arteriosclerosis.

Atherosclerosis develops from cellular and molecular inflammation, a potential factor in sarcopenia (47). Inflammatory factors such as tumor necrosis factor (TNF) and interleukin-6 (IL-6) are essential catabolic factors, which stimulate protein catabolism, inhibit muscle synthesis, and promote muscle atrophy. TNF-α promotes early atherosclerosis by increasing the transcytosis of low-density lipoprotein (LDL) across endothelial cells (48). IL-6 is an upstream regulator and plays a central role in promoting downstream inflammatory response, the leading cause of atherosclerosis. The circulating levels of IL-6 have been independently associated with subclinical atherosclerosis in several studies (49). While other studies have shown that multiple inflammatory factors are negatively associated with muscle mass or muscle strength, elevated cytokines, particularly IL-6, may be a confounding factor in the underlying pathology. This study also found blood pressure, total serum cholesterol, LDL-cholesterol, and high-sensitivity C-reactive protein (hs-CRP) levels in community populations with sarcopenia. Multifactorial analysis showed that independent risk factors for sarcopenia caused serum hs-CRP levels. As a predictor of cardiovascular events, hs-CRP may be related to physical function, and experiments have shown that high CRP levels are related to the risk of losing muscle strength. One of the mechanisms is that high LDL levels in atherosclerosis induce endothelial cells to express CRP, which increases the expression of the endothelial receptor for oxidized low-density lipoprotein (LOX-1) and promotes the occurrence of arteriosclerosis (50). It is speculated that the determination of serum CRP level could be used to assess the risk of arteriosclerosis and screen for skeletal muscle reduction. Oxidative stress is crucial to the pathogenesis of atherosclerosis, and it also plays a vital role in sarcopenia. It was found that disruption of redox homeostasis in motor neurons of Cu/Zn superoxide dismutase knockout mice triggered disruption of neuromuscular junctions, which combats skeletal muscle mitochondrial function and increases the production of reactive oxygen species. When the production of reactive oxygen species in skeletal muscle grows through muscle feedback, the retrograde response is triggered, further impingement of the neuromuscular junction. This vicious circle eventually leads to the breakdown of the neuromuscular junction, denervation and muscle fiber loss, and the occurrence of sarcopenia (51). It can be seen that inflammation and oxidative stress are the common pathogenesis of sarcopenia and atherosclerosis.

### Sarcopenia and CHD

Sarcopenia is closely related to the onset and prognosis of CHD in the elderly and is an independent risk factor for the onset and poor prognosis of CHD in the elderly. At the same time, it is also a risk factor for atherosclerosis in elderly patients and a predictor of poor prognosis for elderly patients with percutaneous coronary intervention (PCI). Studies have extensively shown that sarcopenia may be involved in the development and progression of CHD (3, 30, 52). The study included a total of 475 elderly patients with coronary artery disease who underwent successful PCI to assess sarcopenia by measuring the cross-sectional area of skeletal muscle at the first lumbar vertebra (L1) and exploring the impact of low skeletal muscle mass on the prognosis of patients with coronary artery disease who underwent successful PCI. The results suggest that 29.7% of patients have low L1 skeletal muscle index (SMI). A low L1 SMI is an independent predictor of all-cause mortality and major adverse cardiovascular events (53). A study included a total of 345 Chinese older patients with CHD, with a median age of 74 years. Among the patients, 78 (22.6%) were diagnosed with sarcopenia, according to AWGS. The purpose of this study was to explore the prevalence and prognostic significance of sarcopenia in elderly patients with CHD. The results show that the prevalence of sarcopenia was very high in hospitalized elderly patients with CHD. The incidence of unscheduled follow-up visits in elderly CHD patients with sarcopenia was higher than that in patients without sarcopenia (54). A study used AWGS criteria to assess sarcopenia in 354 elderly patients with CHD over 65 years of age and found that 22.6% were patients with sarcopenia. Its follow-up found that elderly CHD patients with sarcopenia were significantly more than non-sarcopenia patients. The time of no adverse cardiac and brain events was significantly shorter than that of non-sarcopenia patients (54). A study also included 99 patients with acute myocardial infarction with a mean age of 71.6 years. Patients were diagnosed and evaluated for sarcopenia regarding the diagnostic indicators of EWGSOP. The results showed that the prevalence of sarcopenia was 64.6%, which was higher than that of normal people. The prevalence of sarcopenia was much higher in male patients than in female patients, and sarcopenia was associated with thrombolysis-related myocardial infarction scores (55). The study assessed skeletal muscle mass in 378 patients with ST-segment elevation acute myocardial infarction. All-cause death, non-fatal myocardial infarction, non-fatal ischemic stroke, hospitalization for congestive heart failure, and unplanned revascularization were used as the endpoints of long-term follow-up. The results showed that the lower limb skeletal muscle index was still independently associated with the high risk of primary complex events (56). In addition, some studies have shown that muscle mass and muscle strength are negatively correlated with the increase of coronary artery calcification score. Furthermore, muscle mass is positively correlated with coronary artery diastolic ability, suggesting that sarcopenia is related to subclinical coronary atherosclerosis (44). Loss of muscle mass correlates with coronary artery calcification, an independent risk factor for CHD. Decreased skeletal muscle mass also increases the risk of death in patients with CHD (57). Reduced muscle mass diagnosed by CT is a strong predictor of poor prognosis in patients with CHD who undergo percutaneous coronary intervention (53).

The mechanism of the role of sarcopenia in CHD is currently unclear. Studies have shown that sarcopenia and obesity form a vicious circle in the body and then participate in the occurrence and development of CVDs led by CHD through a series of mechanisms such as insulin resistance, mitochondrial dysfunction, oxidative stress, adipokines, and inflammatory factors (58).

## The Pathogenesis of Sarcopenia and CVDs

Sarcopenia is a multi-cause disease with risk factors including lifestyle, changes or imbalances of hormones and inflammatory factors, imbalances of protein synthesis and decomposition, motor unit reconstruction, development, and evolution. Sarcopenia often coexists with CVDs, tumors, chronic non-obstructive disease, chronic kidney disease, endocrine disease, and rheumatic immune disease (59). Various causes of inhibition of skeletal muscle cell proliferation signaling pathways and excessive activation of apoptotic signaling pathways can disrupt the dynamic balance between muscle production and destruction, ultimately leading to diseases (60).

**Figure 1:**
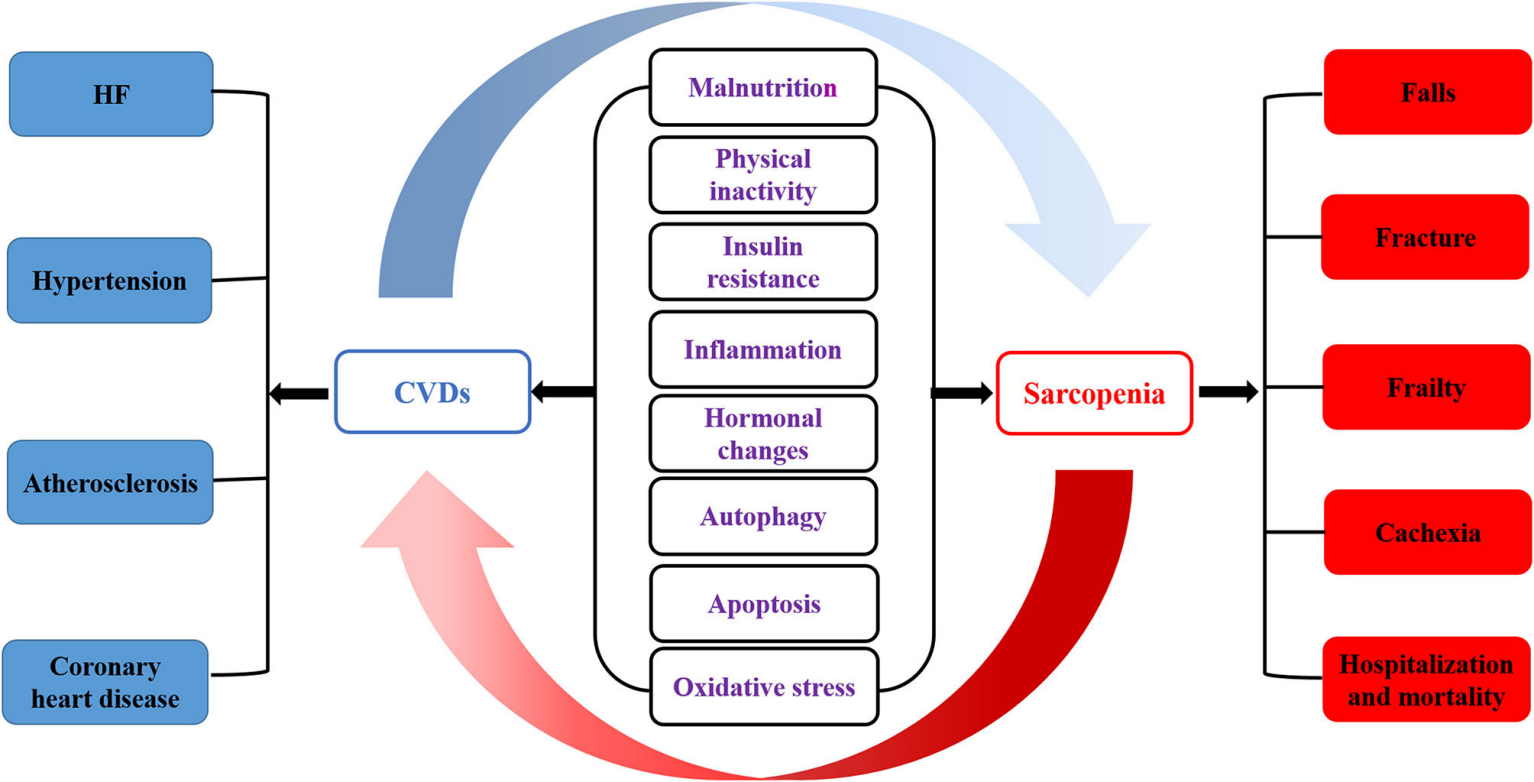
The pathogenesis of sarcopenia and CVDs. Malnutrition, physical inactivity, insulin resistance, inflammation, hormonal changes, autophagy, apoptosis and oxidative stress are involved in the occurrence of CVDs and sarcopenia. Sarcopenia is closely related to cardiovascular disease, which affects each other's course of disease. In addition, CVDs aggravates the adverse outcomes of sarcopenia, including falls, fracture, frailty, cachexia, hospitalization and mortality. At the same time, the prevalence of CVDs in sarcopenia patients is significantly increased, such as HF, hypertension, atherosclerosis and CHD.

Malnutrition, physical inactivity, insulin resistance, inflammation, hormonal changes, autophagy, apoptosis, and oxidative stress are involved in the occurrence of CVDs and sarcopenia (61). Sarcopenia and CVDs are closely related and interact to influence the course of the disease. In addition, CVDs aggravate sarcopenia's adverse outcomes, including falls, fractures, frailty, cachexia, hospitalization, and mortality. At the same time, the prevalence of CVDs in sarcopenia patients is significantly increased, such as HF, hypertension, atherosclerosis, and CHD (Figure 1). HF leads to peripheral ischemia and hypoxia, induces skeletal muscle cell apoptosis, even necrosis, muscle atrophy, and decreases exercise ability; the reduced or lost exercise capacity caused by sarcopenia leads to obesity, dyslipidemia, inflammatory reaction, insulin resistance, and then promotes CVDs (3, 30, 62). The pathophysiological mechanism underlying sarcopenia and CVDs is unclear and progressively focused on and explored by researchers. It was suggested that mechanisms such as inflammation, oxidative stress, and insulin resistance might also be involved in the occurrence and development of CVDs and sarcopenia in the elderly. The mechanism between sarcopenia and CVDs can be understood from the following aspects.

### Inflammation

Studies have shown that long-term systemic chronic inflammation seems to be involved in the whole process of CVDs, and sarcopenia in the elderly (63–65). Senescence-associated secretory phenotype (SASP) is one of the key factors in chronic inflammation-induced atherosclerotic plaque instability, part of the pathogenesis of atherosclerosis (63) and an independent risk factor for myocardial infarction and cardiovascular death (66). As an upstream factor in the inflammatory response, IL-6 reflects the level of systemic inflammation and can prompt the level of systemic catabolism and promote the downstream inflammatory response (67). Studies have confirmed that the long-term activation of the IL-6 signaling pathway is significantly related to the degree of atherosclerosis in elderly patients (49). Selective inhibition of the IL-6 signaling pathway and reduction of systemic inflammatory levels can substantially reduce the incidence of cardiovascular events (68). As individuals age, the body's adipose tissue tends to increase, and levels of free cholesterol and fatty acids rise, which can induce a rise in chronic systemic inflammation by converting M2 macrophages into pro-inflammatory M1 macrophages that produce pro-inflammatory factors such as IL-6 (47). In addition, it was found that the level of IL-6 in patients with sarcopenia is independently related to the occurrence of sarcopenia (47). IL-6 can promote the catabolism of skeletal muscle and cause muscle atrophy. The increase of IL-6 concentration in the blood circulation is related to the severity of HF and the activation of the sympathetic system (69). Inflammation in heart failure patients may promote the development of sarcopenia. The SICA-HF study observed that in patients with HF, IL-6 was significantly higher in the sarcopenia group than in the non-sarcopenia group, but IL-1β and tumor necrosis factor-α did not differ significantly between the two groups (25, 70). Studies have also shown that inflammation activates the body's catabolic pathways, promotes the hydrolysis of muscle protein, leads to an imbalance between protein synthesis and catabolism, and contributes to sarcopenia development (71).

### Oxidative Stress

During the aging process, the body produces large amounts of reactive oxygen species (ROS) due to changes in the function of the respiratory chain; and as the defense function of antioxidant cells is impaired, the ROS produced are not cleared in time and accumulate in the body (72). This is when the body is in a state of oxidative stress. The increased level of oxidative stress in the body can lead to various CVDs such as hypertension, atherosclerosis, myocardial infarction, HF, and arrhythmia (73). The main reasons are as follows: (a) the increase of ROS in the body causes vasoconstriction and promotes arterial hypertension; (b) ROS can negatively affect cardiac calcium processing, cause arrhythmia, and induce hypertrophic signaling and apoptosis to increase cardiac remodeling; (c) ROS has been shown to promote the formation of atherosclerotic plaques; (d) ROS can cause vascular endothelial dysfunction in patients with CVDs and cause adverse cardiovascular events (74–76). Oxidative stress is a common mechanism in many age-related diseases. As we age, the body's antioxidant capacity decreases significantly. The accumulation of ROS in the body will affect the nitrification, nitrosation, carbonylation, and glycation of proteins, thereby affecting muscle protein synthesis (77). At the same time, ROS can also mediate and enhance the hydrolysis of muscle protein, leading to sarcopenia (77). Furthermore, obese patients with sarcopenia have significantly increased levels of circulating oxidative stress and are significantly associated with CVDs risk in such patients (78).

### Insulin Resistance

In recent years, factors related to metabolism have been extensively studied. Insulin resistance is the most representative pathway, and it seems to be related to sarcopenia and CVDs. Many studies have provided reliable clinical evidence, suggesting that insulin resistance is a major cardiovascular risk factor independent of other risk factors in CVDs in older adults in community populations and patients with type I and type II diabetes (79). In patients with ischemic stroke, insulin resistance is independently associated with poor functional prognosis after acute ischemic stroke (80, 81). Skeletal muscle is the leading site of glucose uptake, deposition, and actin secretion, which protect insulin resistance. A reduction in muscle mass can lead to insulin resistance. When the body becomes insulin resistant, on the one hand, insulin secretion in the body is reduced. Glucose homeostasis is disrupted, leading to glucose utilization disorders, while the muscle is an essential organ for the body to absorb and utilize glucose, making its energy supply to muscle significantly reduced; On the other hand, the metabolism of skeletal muscle in limbs of the body increases significantly, and the dysfunction of muscle microvascular function will substantially affect the function and state of skeletal muscles, which will lead to the decrease of skeletal muscle content and sarcopenia (82–84).

## Joint Intervention of Sarcopenia and CVDs

**Figure 2:**
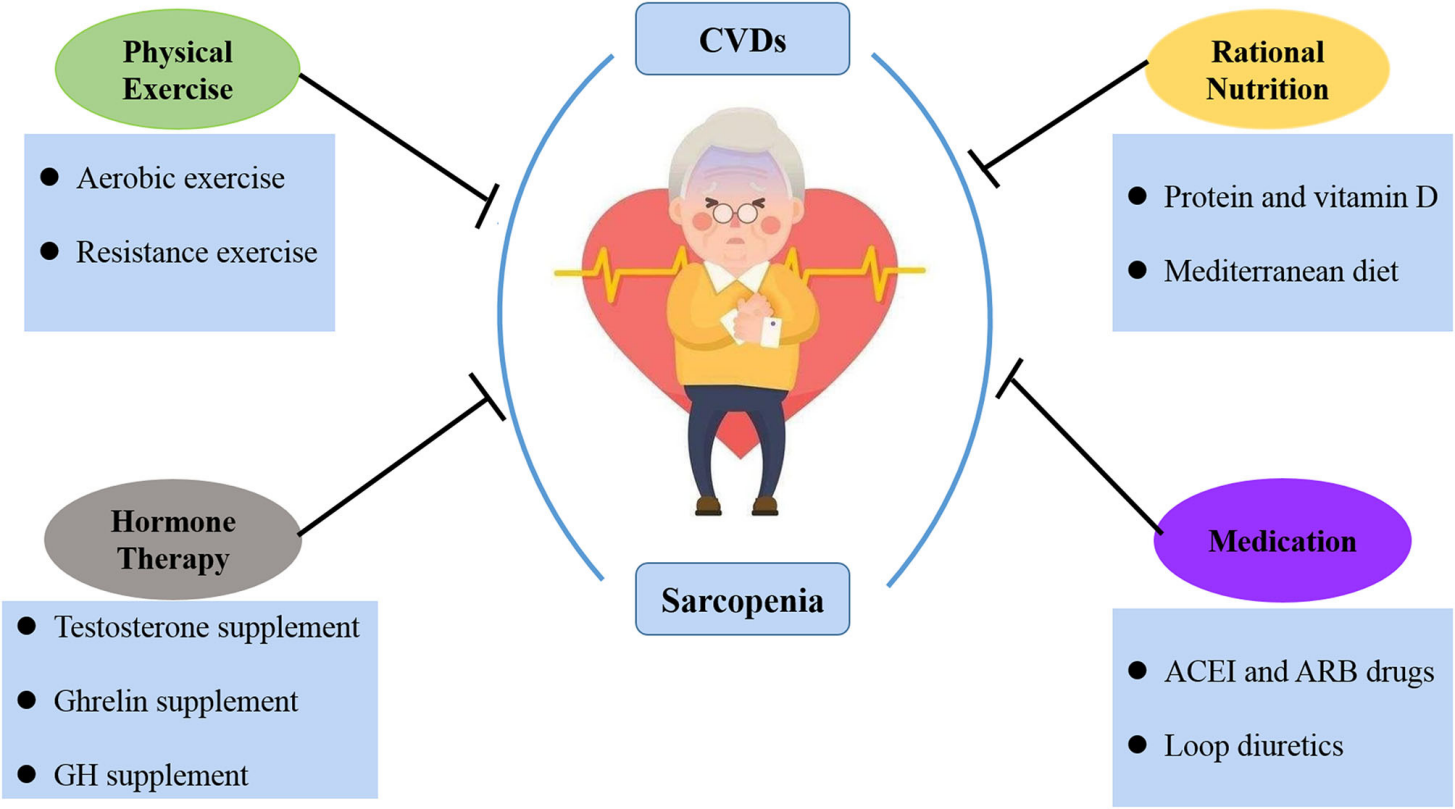
The treatment of sarcopenia and CVDs. At present, the joint intervention of sarcopenia and CVDs is mainly from physical exercise, rational nutrition, hormone therapy and medication.

Currently, the central combined interventions for sarcopenia and CVDs come from physical exercise, proper nutrition, hormone therapy, and medication (Figure 2).

### Physical Exercise

Physical exercise is an economical, safe, and effective intervention for both CVDs and sarcopenia. Physical exercise methods include active and passive exercise and aerobic and resistance exercise, increasing muscle mass and strength, improving exercise and balance ability, and reducing falls and fractures (52, 85–88). Aerobic exercise can be carried out with simple equipment such as swimming, jogging, and push-ups or with the aid of elastic bands. These exercises effectively prevent CVDs that may be caused by an immediate increase in heart rate and blood pressure (89). Resistance exercise can increase muscle volume and muscle contractility and improve submaximal exercise endurance in patients with heart failure. Studies have shown that a combination of aerobic and resistance exercise can increase the effectiveness of exercise rehabilitation. Medium to long-term resistance training, aerobic exercise, and other forms of exercise or a mixture of exercise can significantly improve muscle strength, increase muscle mass, slow the progression of HF and inhibit skeletal muscle breakdown in some patients with sarcopenia (90). In one study, patients with chronic heart failure were found to have significantly improved endurance and 6-min walking distance in all muscle groups after 10 weeks of high-intensity progressive resistance exercise (91). Recent studies have further confirmed the importance of exercise training in suppressing inflammatory factors, reducing oxidative stress, reducing myostatin expression, and inhibiting the ubiquitin protease system in patients with HF (92).

### Rational Nutrition

Nutritional intervention is currently the primary prevention and treatment method for sarcopenia, including supplementation of protein and amino acids (β-Hydroxy-β-Methyl butyrate (HMB), antioxidants, long-chain fatty acids, vitamin D, and creatine) (93, 94). Protein and vitamin D intake is crucial to the prevention and treatment of sarcopenia. Protein is essential for muscle metabolism in the body. Essential amino acids such as leucine and isoleucine are necessary to promote muscle protein synthesis (95, 96). There are still controversies regarding vitamin D supplementation for the prevention and treatment of sarcopenia. The correlation between vitamin D level and muscle mass is poor, but it can slightly improve muscle strength (97, 98). In addition, HMB is the active metabolite of the essential amino acid leucine and has a critical interventional effect on sarcopenia. It has been shown to inhibit muscle proteolysis, promote muscle protein synthesis, inhibit muscle protein decomposition, maintain cell membrane integrity, improve immunity and reduce inflammation. A meta-analyses systematic study showed that nutritional supplementation with HMB can enhance lean muscle mass and preserve muscle strength and function in the elderly with sarcopenia or frailty (99). A review of Clinical Trials showed that HMB supplementation is essential for the maintenance of muscle mass in the elderly over 65 years old, especially the elderly who are bedridden or sedentary, and contributes to the reduction of muscle metabolism. Many studies have shown that HMB increased muscle mass and strength in older people with reduced lean body mass (100–102). In addition, studies have shown that HMB supplementation has a positive effect on lowering plasma cholesterol and blood pressure, thereby reducing the risk of cardiovascular disease (103–105). It was shown that HMB slows HF progression by maintaining lean body mass and limiting the effects of cachexia. Therefore, HMB is likely to be crucial for the nutritional management of patients with HF-induced cachexia (106). Therefore, nutritional support is essential for the recovery of sarcopenia patients. The Mediterranean diet is an ideal diet for patients with CHD, rich in nutrients and balanced. The Mediterranean diet also helps delay muscle wasting in the elderly and reduces the risk of sarcopenia (107). As with exercise, patients need to adhere to an appropriate diet for a long time to achieve good outcomes.

### Hormone Therapy

Some studies have shown that supplementing testosterone is beneficial to muscle and skeletal tissues (108, 109), particularly in increasing muscle strength, improving mobility, and reducing the hospitalization rate of elderly patients with sarcopenia (110). Decreased testosterone can cause fatigue and weakened exercise capacity, while testosterone supplementation can increase muscle strength and improve exercise capacity (111). At lower doses, testosterone increases protein synthesis, thus increasing muscle mass (112). In comparison, testosterone activates the recruitment of satellite cells at higher doses and reduces adipose-derived stem cells, thereby increasing myogenesis and reducing adipogenesis (110). Testosterone replacement therapy can improve metabolism and exercise tolerance in patients with chronic heart failure. Results showed an increase in peak oxygen uptake, 6-min walk distance, and body weight in the treatment group compared to the control group, directly related to the serum testosterone concentration (113). However, testosterone therapy may increase the risk of benign prostatic hyperplasia and tumor in male patients and masculinize female patients, limiting its wide clinical application.

Ghrelin exerts protective effects in skeletal muscle by regulating autophagy, apoptosis, insulin resistance, and inflammation (114). Ghrelin can also inhibit atherosclerosis, ischemia-reperfusion injury, ventricular remodeling, and improve cardiac function and endothelial function (115). As ghrelin is highly expressed in tumor tissue, its clinical application needs careful evaluation. However, attention should be paid to the side effects of testosterone therapy, such as benign prostatic hyperplasia, prostate cancer, polycythemia, and sleep apnea syndrome. Moreover, the intramuscular injection has higher safety than oral treatment.

Growth hormone (GH) is an essential endogenous hormone that can promote the growth of organs and tissues, promote protein synthesis, and affect fat and mineral metabolism. GH is involved in the regulation of skeletal muscle growth mainly through insulin growth factors. It can increase skeletal muscle mass but has no noticeable effect on muscle strength (116). Notably, GH can increase the risk of fluid retention and insulin resistance and adversely affect the cardiovascular system.

### Medication

Angiotensin-converting enzyme inhibitors (ACEI) and angiotensin receptor blockers (ARB) have multiple cardiovascular protective effects, and their anti-inflammatory and antioxidant effects also benefit muscle tissue (117). Early studies have found that ACEI drugs can delay the decline of muscle mass. Recent studies have negated its effect on muscle mass and muscle strength. However, ARB can effectively improve the muscle strength of hemodialysis patients (118). In addition, recent studies have found that loop diuretics can increase the risk of sarcopenia in non-dialysis patients with chronic kidney disease. In patients with HF, spironolactone can prevent skeletal muscle loss and improve muscle strength, possibly due to improved cardiac function (119).

## Shortcomings and Prospects

Globally, t the incidence of sarcopenia is gradually increasing, and it has received full attention from European and American countries. However, for the Asian region, the research on sarcopenia is still in its infancy. As for sarcopenia, from the initial focus on muscle mass to the latest 2018 EWGSOP2, muscle strength is the primary diagnostic element, indicating that the understanding of its essence is constantly deepening. However, many areas still need to be further explored, including the pathophysiological processes such as the occurrence, development, and outcome of sarcopenia, sarcopenia-related biomarkers, screening, and preventive measures for high-risk people. In terms of the diagnosis of sarcopenia, there are some subjective diagnosis critical values at present. More objective and reasonable diagnosis critical value needs to be determined by standardized clinical research big data and gender and regional specificity. In terms of treatment, it is considered that nutrition and exercise are two treatment methods that can be implemented clinically to delay sarcopenia. However, the specific application, usage, dosage, and effectiveness of related nutritional supplements in nutritional therapy still need more research data to support. The exercise therapy method, frequency, and intensity also need clinical research to further confirm and refine. Although many studies have shown that nutritional s supplements combined with exercise are effective in treating sarcopenia, more research is also needed to standardize the treatment plan. At present, in terms of drug treatment, there is still a lack of clinical first-line drugs, and a small number of drugs for the treatment of sarcopenia are expected to enter phase III clinical trials in the next few years. However, the preliminary research of many drugs will face significant challenges.

Sarcopenia needs more basic and clinical research to explore its risk factors, pathogenesis, and intervention measures. At present, there is no unified conclusion on the mechanism of the relationship between sarcopenia and CVDs. However, according to the existing research, it can be determined that there are many similar pathophysiological mechanisms between sarcopenia and CVDs. Furthermore, sarcopenia has a specific correlation with the poor prognosis of CVDs. Therefore, it is necessary to pay attention to the common pathway of the two diseases, carry out systematic, basic, and large sample clinical research, and look for reliable biomarkers, so as to provide new ideas for the prediction and diagnosis of sarcopenia and cardiovascular diseases, as well as the early intervention of adverse prognosis.

## Conclusion

In summary, sarcopenia and CVDs are highly prevalent in the elderly and share common pathogenesis and interactions. Understanding their relationship is still in its initial stages, and more clinical and experimental data are needed. A large number of studies have shown that the progression of CVDs and the decline in muscle function will further worsen the patient's condition. By screening patients for sarcopenia at an early stage, establishing effective early detection methods and evaluation methods, and providing early and comprehensive interventions, the progression of the disease can be effectively delayed. Nevertheless more importantly, patients with CVDs should be rehabilitated as soon as possible to break the vicious cycle of sarcopenia and CVDs through scientific nutritional programs and training guidance. Effective treatment of either sarcopenia or CVDs can have a positive impact on another disease. However, some drugs have acted as a double-edged role in the treatment of the two diseases. A healthy lifestyle and proper drug treatment have become necessary means for preventing and treating CVDs and sarcopenia. In the future, more high-quality research is still needed to provide a basis for optimal treatment options for people with specific diseases, such as CVDs co-morbid with sarcopenia.

## Author Contributions

NH drafted the manuscript of this review article. SZ and HY conceived and supervised the manuscript. YZ and LZ also collected and organized the information and prepared the table and figures for the manuscript. All authors contributed to the article and approved the submitted version.

## Funding

This study was funded by the Ningbo Health Branding Subject Fund (PPXK2018-01), Ningbo Medical Science and Technology project (2016Z01), Zhejiang Provincial Public Service and Application Research Foundation, China (LGF20H250001), and Ningbo HwaMei Research Fund (2019HMZD09).

## Conflict of Interest

The authors declare that the research was conducted in the absence of any commercial or financial relationships that could be construed as a potential conflict of interest.

## Publisher's Note

All claims expressed in this article are solely those of the authors and do not necessarily represent those of their affiliated organizations, or those of the publisher, the editors and the reviewers. Any product that may be evaluated in this article, or claim that may be made by its manufacturer, is not guaranteed or endorsed by the publisher.
